# Predictive Factors and Interventional Modalities of Post-stroke Motor Recovery: An Overview

**DOI:** 10.7759/cureus.35971

**Published:** 2023-03-10

**Authors:** Ahmad S Badawi, Ghazi H Mogharbel, Sultan A Aljohani, Amal M Surrati

**Affiliations:** 1 Medicine and Surgery, Taibah University, Medina, SAU; 2 Family and Community Medicine, Taibah University, Medina, SAU

**Keywords:** motor impairment, post-stroke recovery, rehabilitation, motor recovery, stroke

## Abstract

Stroke is the most common cause of motor impairment worldwide. Therefore, many factors are being investigated for their predictive and facilitatory effects on recovery of motor function after stroke. Motor recovery can be predicted through several factors, such as clinical assessment, clinical biomarkers, and gene-based variations. As for interventions, many methods are under experimental investigation that aim to improve motor recovery, including different types of pharmacological interventions, non-invasive stimulation, and rehabilitation training by inducing cortical reorganization, neuroplasticity, angiogenesis, changing the levels of neurotransmitters in the brain, and altering the inflammatory and apoptotic processes occurring after stroke. Studies have shown that clinical biomarkers combined with clinical assessment and gene-based variations are reliable factors for predicting motor recovery after stroke. Moreover, different types of interventions such as pharmacological agents (selective serotonin reuptake inhibitors {SSRI}, noradrenaline reuptake inhibitors {NARIs}, levodopa, and amphetamine), non-invasive stimulation, and rehabilitation training have shown significant results in improving functional and motor recovery.

## Introduction and background

Stroke is a medical emergency. It is the leading cause of disability worldwide, and one of its most impairing complications is motor weakness in one or more parts of the body [1]. In addition, it is considered one of the leading health problems in the Middle East, with an incidence rate of 29.8 out of 100,000 people in the Kingdom of Saudi Arabia to 57 per 100,000 in Bahrain. The 28-day mortality rate of stroke differs across the Middle East, ranging from 10% in Kuwait to 31.5% in Iran [2]. In the United States, approximately 800,000 people are affected by stroke annually, with most of the cases being primary stroke. Costs of stroke in the United States, whether direct or indirect, were estimated to be 68.9 billion in 2009 [3]. We reviewed the literature on motor recovery because the psychological, social, and physical well-being of a person depends significantly on his ability to carry out day-to-day motor activities and their ability to interact with others. Furthermore, clinicians design suitable treatment plans according to the available information regarding possible interventions and factors contributing to the prediction of motor recovery [1].

As proven in earlier studies, patients with upper extremity (UE) paresis are disabled to different degrees than patients with lower extremity (LE) paresis, and are treated with different measures [4]. New treatments are being developed because of the ongoing exploration of the mechanisms underlying post-stroke functional recovery [5]. Moreover, several factors have been hypothesized to play an important role in the development of post-stroke motor impairment and subsequent recovery, such as socioeconomic factors, genetic factors, and other clinical factors impacting motor recovery. As the rate of stroke survivors is increasing due to advanced medical technologies, proper knowledge about motor recovery and possible interventions to facilitate recovery is widely demanded [6].

We are reviewing this topic to provide an overview of the possible predictive methods and treatment modalities to facilitate motor recovery after stroke so that clinicians and researchers can further investigate these modalities. We searched for articles, mainly in Google Scholar and PubMed. We included the most updated articles on a certain topic with the most reliable study design. Articles that were outdated or the ones that were investigated with a better and more reliable study design were excluded. We aim to identify the different factors contributing to the prediction of post-stroke motor recovery and to discuss the available interventional modalities for stroke patients to facilitate motor recovery.

## Review

Prediction of motor recovery

Clinical Assessment and Biomarkers

**Figure 1:**
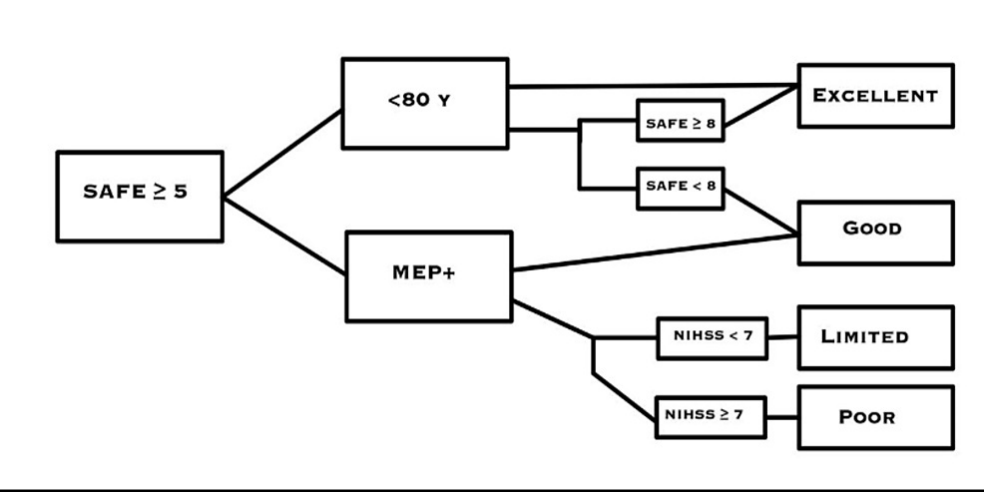
PREP2 algorithm to predict upper limb functional outcome three months after stroke This figure was obtained from Connell et al. [11]. PREP: Predict REcovery Potential, SAFE: shoulder abduction and finger extension, MEP: motor evoked potential, NIHSS: national institutes of health stroke scale.

The prediction of post-stroke motor recovery by clinical assessment alone is difficult, but helpful in the prediction of the following resolution [7]. Researchers have started to define the assessment parameters that help predict post-stroke motor recovery and have defined some of them. First, blood markers could provide beneficial information for improving the prediction of post-stroke physical recovery [8]. In addition, a combination of clinical assessment and local diffusion homogeneity (LDH) is used to predict post-stroke motor recovery. The use of a combination of clinical assessment and LDH was found to be accurate in predicting upper extremity disability recovery within 12 weeks of subcortical infarction [9]. Furthermore, the combination of clinical assessment and the Predict REcovery Potential (PREP)2 algorithm was useful in predicting upper extremity recovery three months after stroke, with the algorithm achieving accurate predictions in 75% of patients [10]. A combination of age, shoulder abduction, finger extension (SAFE), motor evoked potentials (MEP), and National Institutes of Health Stroke Scale (NIHSS) have been used to produce the analysis shown in Figure 1 [11]. Stroke also causes changes in the structure of the cortico-spinal tract (CST); therefore, a study aiming at determining the relationship between fractional anisotropy (FA) and motor outcomes was done. The results showed that CST FA could enhance motor outcome prediction up to 84.6% based on early motor scores, age, and level of education [12].

Gene-Based Variations

Genetic differences among people are another factor that affects motor implications of strokes and are linked to the genome-wide association (GWA) approach. Genetic variations are associated with the type of stroke (cortical/subcortical, white, or grey matter) influencing the recovery process and its possibility [13]. Genetic polymorphisms were also found to affect the levels of brain-derived neurotrophic factor (BDNF), dopamine, and apolipoprotein E (APOE), which in their turn affect neuroplasticity, functional recovery, and the type of stroke [14]. APOE ε2/ε3/ε4 and BDNF val66met polymorphisms were found to exist in patients with poorer motor outcomes than those without their presence, coexisting with cognitive impairment in patients with BDNF val66met polymorphism [15,16]. Therefore, polymorphic differences and the subsequent involvement of chemical factors may be linked to how well a patient responds to rehabilitation training, and can be a target for the development of pharmacological interventions [13,14]. It has been established that people with the APOE polymorphism are approximately half as likely as those without it to have minor or no disabilities (18.2% vs. 35.5%) [15].

Interventions for motor recovery

Pharmacological Interventions

Proofs of pharmacological agents that are effective in the modulation of neuronal reorganization and promotion of somatosensory representations are emerging. Several studies (human and animal studies) examined the effects of several agents, including growth factors and small and large molecules. Selective serotonin reuptake inhibitors (SSRI), such as fluoxetine, reboxetine, or citalopram, have been found to be effective in modulating different aspects of motor recovery and the probability of promoting angiogenesis by upregulation of vascular endothelial growth factor (VEGF) in many areas of the brain [17]. VEGF was additionally found to be acting as a neurogenetic and neuroprotective factor and it assists in reducing the infarcted size of brain tissue in rat experiments. Results also showed that it decreases the size of infarct by approximately 30% in rats [18]. Moreover, other studies investigating the administration of amphetamine with mild physical exercise in a group of patients found that it increased the extent of motor recovery and rate of neuroplastic processes [19]. Noradrenaline reuptake inhibitors (NARIs) are also a possible choice. They have been shown to improve simple hand movements and the frequency of finger tapping in patients with chronic stroke by modulating ischemic neuronal networks, as well as the elevation of neuronal coupling ipsilateral to the lesion [20]. In addition to the previously mentioned drugs, dopamine precursors such as levodopa (LD) increase the sufficiency of physical exercise and show clinically significant improvements in motor function in hemiplegic patients compared to physiotherapy alone. It was established in a study that it improved motor recovery in patients with primary stroke after three weeks of levodopa administration by 6.4 points compared to the placebo group (4.1 points) [21]. While many groups, such as granulocyte-colony stimulating factors and cholinergic groups, have yielded promising results, they need further studies before being officially applicable [22]. 

Non-invasive Stimulation

Repetitive transcranial magnetic stimulation (rTMS): In a study done recently, it has been shown that stimulation with different rTMS frequencies in both motor cortices can enhance the behavior of the paretic arm. They investigated whether self-movement could be promoted many years after stroke by rTMS, and which hemisphere stimulated by rTMS (affected or intact) would be the ideal place for spasticity attenuation and movement development in the weakened arm. In this study, 64 patients were recruited and divided into four groups. The results showed that spasticity could be modified by stimulating either the affected hemisphere or the intact hemisphere, but motion can be induced only by stimulating the intact motor path and its surrounding area. Weakened extremities are shown to be improved by rTMS even a long time after an encounter with a stroke when traditional rehabilitative methods are not applicable [23]. A recent study indicated that rTMS enhanced rehabilitation of extremity motor function and changed the degree of cortical excitability. Therefore, rTMS may be the best intervention for patients with early and pure subcortical stroke. In terms of various stimulation parameters, the number of stimulation sessions is proportional to the effect of rTMS [24].

Transcranial direct current stimulation (tDCS): tDCS is a secure, non-invasive brain stimulation method that can remodel the excitability of selected brain regions by modifying the neuronal membrane potential depending on the current polarity transmitted through the scalp by placing electrodes. Anodal stimulation increases cortical stimulation, whereas cathodal stimulation reduces cortical stimulation. tDCS has extremely high clinical potential for use in both acute and chronic stroke recovery. Therefore, it has become an important adjuvant treatment when combined with other stimulation techniques [25]. The results of a recent study published in 2018 showed that tDCS could modulate cortical excitability with a durable after effect. By increasing synaptic plasticity requiring BDNF secretion and TrkB activation, tDCS may enhance motor skill learning. Several other tDCS studies have investigated the possible beneficial outcomes and safety profiles of post-stroke motor recovery [26]. 

Electrical stimulation: Functional electrical stimulation is a useful method for motor recovery in stroke patients. Several studies have shown that motor function, quality of life, and gait are enhanced by functional electrical stimulation. Application of the technique in more than one group of muscles and activation of impulses through active movements. The results demonstrated that functional electrical stimulation by itself did not show more promising results than other conventional physiotherapy techniques; however, when combined with other interventions, it was very helpful [27]. Neuromuscular electrical stimulation (NMES) has also been used to decrease spasticity and enhance the range of motion in stroke patients. Additionally, NMES can be classified as a treatment option in combination with other intervention modalities to improve spasticity and range of motion in post-stroke patients [28].

Optogenetic stimulation: Post-stroke optogenetic stimulation improves neurovascular coupling and recovery. Results illustrated an improvement in motor function after optogenetic stimulation of the contralesional lateral cerebellar nucleus (cLCN) and systemic neuronal nitric oxide synthase (nNOS) inhibition, but it also suggested that nNOS might have a maladaptive effect in post-stroke recovery [29]. 

Targeted vagus nerve stimulation (VNS): Combined with rehabilitation, VNS improves plasticity and supports recovery of upper limb function after stroke. Vagus nerve stimulation increases the production of neuromodulators, such as acetylcholine and norepinephrine, which improves plasticity. Also, the combination of VNS with rehabilitation training enhances functional motor recovery; this is related to the reorganization of the synaptic motor networks controlling the impaired limb [30]. 

Rehabilitation Training

Conventional physiotherapy: Conventional physiotherapy is one of the oldest used interventions for those with a disabled limb or part of the body. Physical exercise has been shown to reduce inflammation and apoptosis, increase growth factors, promote angiogenesis, and stimulate paralyzed muscles. However, it may exacerbate sensorimotor deficits according to the chosen exercise. Nevertheless, evidence suggests that these conventional physical interventions should be performed in conjunction with pharmacologically based interventions [31]. An earlier study has also mentioned the result of increased walking speed and balance and increased sufficiency of arm function upon continuing conventional physiotherapy [32]. Moreover, early additional physiotherapy (AED) showed helpful results in improving patients’ independence [33]. 

Mirror therapy: Mirror therapy (MT), which involves placing a mirror on the side of the paretic leg to reflect the movement of the normal leg, is an emerging type of intervention that shows promising results in improving motor recovery after stroke. Mirror therapy assists in improving upper extremity motor function, motor impairment, day-to-day activity performance, and pain sensation [34]. Nevertheless, MT was effective in cortical reorganization and increased activity in brain areas responsible for body part awareness, such as the posterior cingulate gyrus and precuneus, which may increase the sense of the paralyzed limb and therefore decrease the learned non-use phenomenon of the limbs [35]. MT was also suggested to have a synergistic effect with NMES and they illustrated enhanced hand movement recovery more than any of them alone, a combination that resulted in an increase in Fugl-Meyer scores and a considerable increase in hand extension power [36].

Dysphagia rehabilitation: Dysphagia is one of the main complications encountered by patients after stroke, and since it increases the risk of several unwanted problems such as malnutrition and aspiration, aiming to resolve it should be a main desired outcome. Since it is asymmetrically represented in the cortex, stroke affecting the dominant hemisphere results in a severe impairment of swallowing function. One method that has shown positive results is rTMS, which augments swallowing coordination and reduces the reaction time of liquids when swallowing [37]. A recent study also studied the effect of chair-stand exercise on dysphagia and concluded that it improves dysphagia as well as activities of daily living [38]. Lingual exercises were also examined, which play a role in strengthening the tongue with a subsequent improvement in swallowing [39]. 

Visual reality-based interventions: Virtual reality is a new technique that is evolving and becoming increasingly significant, and many studies are discovering its health benefits. Visual reality improves outcomes in post-stroke patients and has a significant advantage compared to conventional therapies. Moreover, it assists in improving finger fractionation, range of finger movement, and finger speed [40]. Upon looking for gait improvement, visual reality-based games helped patients refine their balance [41]. 

Music-based intervention: Music-based therapy is beneficial, especially in patients with left hemispheric stroke, and is a prolific field due to the ongoing exploration of the neural circuity of brain areas, which helps left hemispheric stroke patients regain their speech or even improve it. Evidence suggests that speech areas (located in the left hemisphere) and singing areas are different, suggesting that the speech area is localized and concentrated at a point where singing areas are somewhat diffuse. This study also reported that neuroplasticity can be efficiently induced by music therapy in patients after a left hemispheric stroke. In addition, the element of rhythm present in music, along with melody, contributes to the mechanism underlying speech recovery. Rhythmic intonation, which is slow, is mainly processed by the right hemisphere as it processes slow neuronal signals, while music is processed by the right hemisphere, a process that helps bypass the stroked left hemisphere. Nonetheless, left-hand tapping activated the right cerebral hemisphere, resulting in increased activation. Therefore, important clinical evidence reinforced by neuroimaging of neuronal circuity suggests the effectiveness of music-based therapy in improving speech and language and retraining the brain in post-stroke patients [42].

Robot-assisted rehabilitation: Robot-assisted rehabilitation is another method that is currently being evaluated. Primary studies suggest that post-stroke robot-assisted rehabilitation yields results similar to conventional physical therapy regarding movement, walking ability, and day-to-day activities. However, for patients with severely impaired lower limbs, robot-assisted rehabilitation showed better results than regular physiotherapy. The treatment sustainability of limbs showed better results in patients with robot-assisted interventions, though these results require further investigation. Treatment sustainability of daily activities showed no difference between the methods [43]. 

Other Interventions

Paired associative stimulation (PAS) is a technique used to improve functional motor recovery after stroke. Results show potential evidence that PAS increases the degree of cortical excitability, which could favorably affect motor function in stroke patients [44]. In addition, strength training (ST), an effective and noninvasive intervention, can be used for the recovery of motor function. Results show that progressive resistance training is the most effective intervention to improve strength when properly targeted [45]. Finally, vibrotactile glove rehabilitation has a potential effect on motor function, especially in patients in the recovery plateau, by increasing motivation [46]. 

## Conclusions

Through our literature review, we found that the prediction of post-stroke motor recovery using clinical assessment is difficult, but a combination with biomarkers helps in predicting motor recovery more accurately. Genetic variations and genetic polymorphic differences among populations are also suggested to play a role in predicting motor recovery after stroke. Pharmacological interventions, MT, visual reality-based interventions, robot-assisted interventions, music-based therapy, and several dysphagia rehabilitation methods have shown interesting results in improving post-stroke motor recovery. Other noninvasive interventions, such as rTMS, tDCS, VNS, NMES, ST, and optogenetic stimulation, are useful in modulating motor recovery after stroke.
